# Comparing different wavelet transforms on removing electrocardiogram baseline wanders and special trends

**DOI:** 10.1186/s12911-020-01349-x

**Published:** 2020-12-30

**Authors:** Chao-Chen Chen, Fuchiang Rich Tsui

**Affiliations:** 1 Tsui Laboratory, Department of Biomedical and Health Informatics, Children’s Hospital of Philadelphia, Philadelphia, PA USA; 2 Department of Biomedical Engineering, National Cheng-Kung University, Tainan, Taiwan; 3 Department of Anesthesiology and Critical Care, Perelman School of Medicine, University of Pennsylvania, Philadelphia, PA USA

**Keywords:** Baseline wander, Electrocardiogram, Wavelet transform, Mean-square-error

## Abstract

**Background:**

Electrocardiogram (ECG) signal, an important indicator for heart problems, is commonly corrupted by a low-frequency baseline wander (BW) artifact, which may cause interpretation difficulty or inaccurate analysis. Unlike current state-of-the-art approach using band-pass filters, wavelet transforms can accurately capture both time and frequency information of a signal. However, extant literature is limited in applying wavelet transforms (WTs) for baseline wander removal. In this study, we aimed to evaluate 5 wavelet families with a total of 14 wavelets for removing ECG baseline wanders from a semi-synthetic dataset.

**Methods:**

We created a semi-synthetic ECG dataset based on a public QT Database on Physionet repository with ECG data from 105 patients. The semi-synthetic ECG dataset comprised ECG excerpts from the QT database superimposed with artificial baseline wanders. We extracted one ECG excerpt from each of 105 patients, and the ECG excerpt comprised 14 s of randomly selected ECG data. Twelve baseline wanders were manually generated, including sinusoidal waves, spikes and step functions. We implemented and evaluated 14 commonly used wavelets up to 12 WT levels. The evaluation metric was mean-square-error (MSE) between the original ECG excerpt and the processed signal with artificial BW removed.

**Results:**

Among the 14 wavelets, Daubechies-3 wavelet and Symlets-3 wavelet with 7 levels of WT had best performance, MSE = 0.0044. The average MSEs for sinusoidal waves, step, and spike functions were 0.0271, 0.0304, 0.0199 respectively. For artificial baseline wanders with spikes or step functions, wavelet transforms in general had lower performance in removing the BW; however, WTs accurately located the temporal position of an impulse edge.

**Conclusions:**

We found wavelet transforms in general accurately removed various baseline wanders. Daubechies-3 and Symlets-3 wavelets performed best. The study could facilitate future real-time processing of streaming ECG signals for clinical decision support systems.

## Background

Artifacts are common in ECG recording and they may cause interpretation difficulty or inaccurate analysis, especially in real-time ECG data processing. Baseline Wander (BW) is one of severe artifacts that could cause difficulty of diagnosis [1]. For example, BW might affect the accuracy of measuring the elevation or depression of ST-segment, which serves as a critical clinical feature to early detect patients’ diseases [2]. Thus, Baseline Wander removal is one of imperative ECG preprocessing steps. There are many methods to remove BW, such as Band-pass filter [3], interpolation [4], etc. Among these methods, wavelet transform (WT) have the best result given its nature of addressing both temporal and frequency changes of a signal [5]. Nevertheless, there is a lack of research that systematically evaluates ECG baseline wander removal using different wavelet transforms. This study aimed to evaluate various wavelet transforms and identify best wavelet transforms for removing ECG baseline wander effect. The outcomes of this study could facilitate future real-time processing of streaming ECG signals.

## Methods

**Fig. 1:**
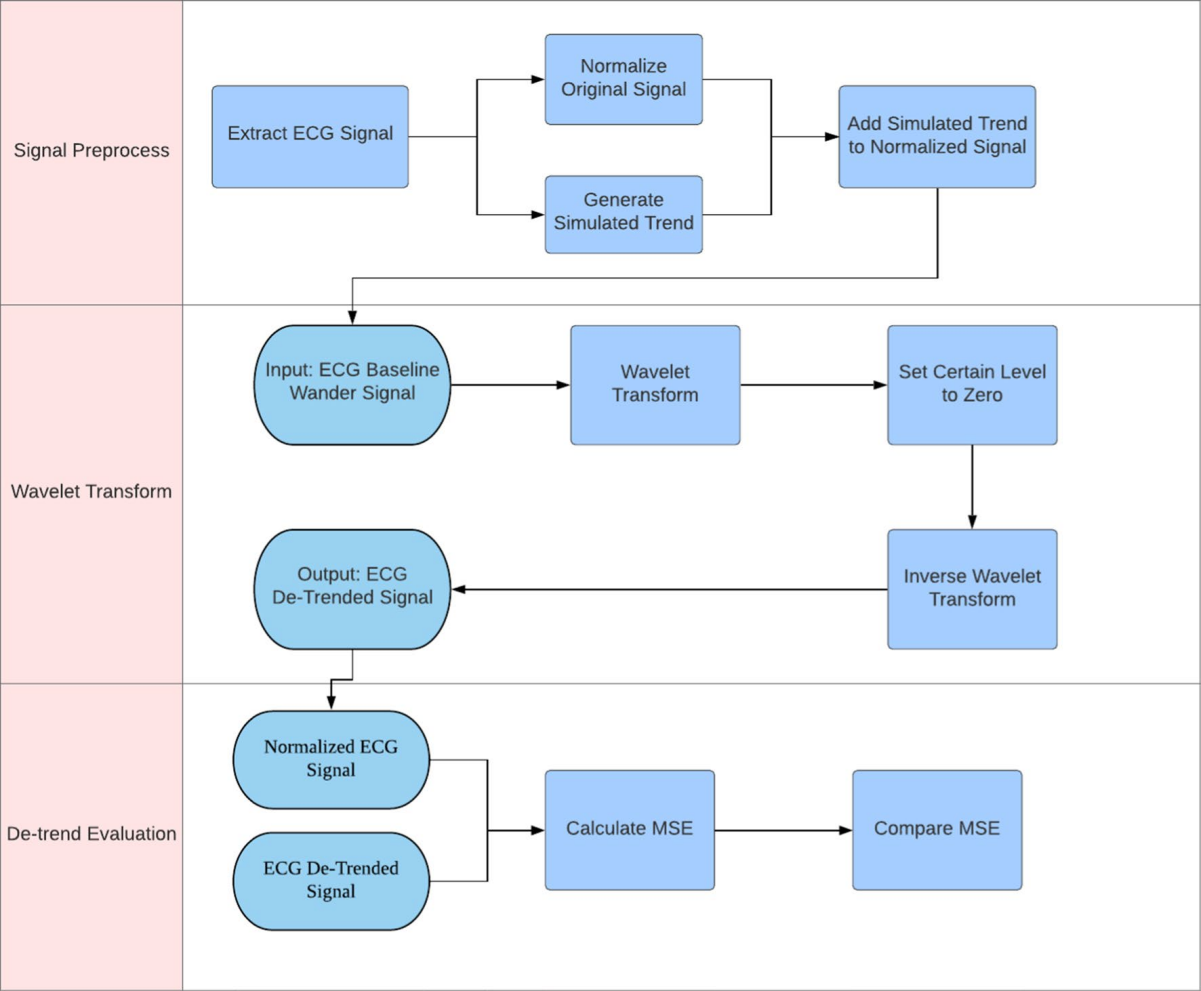
Information flow of the study with three stages. The first stage is signal processing, which formed semi-synthetic data by superimposing a normalized raw ECG signal with an artificial baseline wander (BW or trend). The second stage is wavelet transform (WT) and BW removal. The third stage is WT evaluation, which measured the mean square errors between the normalized raw ECG data and the de-trended semi-synthetic ECG signal

This study was approved by the Institutional Review Board at the Children’s Hospital of Philadelphia. We first described our research dataset, artificial baseline wanders, wavelet transform families, followed by evaluation approach. Figure 1 summarizes the information flow of this study.

### Dataset

In this study, we used publicly available QT database in Physionet [6] that comprises a total of 105 patients and each patient had two ECG channels with 15-min excerpts of recordings. The sampling rate of ECG signal was 250 Hz. We randomly extracted 14 s of ECG data from one of the two channels in each patient, which generated 105 14-s excerpts of ECG data. When the QT database was initially created in the Physionet, all the ECG data in the database was manually selected to minimize the effects of significant baseline wander and other artifacts [7].

After randomly extracting a 14-s episode of ECG data, we further normalized the data based on Eq. 1; thus, all the ECG episodes were in the same range to avoid potential signal strength (amplitude) bias.

1 $${x}_N\left[t\right]=\frac{x\left[t\right]-{x}_{min}}{x_{max}-{x}_{min}},$$

where *x*_*max*_ and *x*_*min*_ were the maximum and minimum values during the 14 s and *t* represented time.

### Simulated (artificial) baseline wanders (trends)

To simulate baseline wanders, we created 10 sinusoidal waves ranging from 0.05 Hz (20-s cycle) to 0.5 Hz (2-s cycle) and − 1 mV to 1 mV and we formed a synthetic ECG data by superimposing the artificial trend (with the same sampling rate 250 Hz in the raw ECG data) to an extracted ECG data from the QT database. In addition, we created two additional types of special trends, i.e. a step function and a spike function, to simulate real-world voltage spikes. We created a total of 12 trends (10 sinusoidal waves and 2 special trends). Equation 2 represents the semi-synthetic ECG data, y[t]:

2 $$y\left[t\right]={x}_N\left[t\right]+w\left[t\right],$$

where *w[t]* represents an artificial baseline wander (or trend).

### Wavelet transforms

Unlike Fourier transform in signal processing that represents a temporal signal solely in frequency domain, a wavelet transform (WT), represents a temporal signal in both time and frequency domains using finite support basis functions (e.g., wavelet) in different resolutions (levels or frequency bands). We chose 5 commonly used wavelet transform families with a total of 14 wavelets: Daubechies (dbN, where N ∈{ 1,2,3,4}), Coiflets (coifN, where N ∈{ 1,2,3,4}), Symlets (symN, where N ∈ {3,4,6,10}), Fejer-Korovkin (fkN, where *N* = 4), and Meyer (dmey) [8]. The N in each wavelet family represents the number of vanishing moments [8]. For each WT, we performed wavelet transform of an input (ECG) signal up to 11 levels (the relationship between sampling frequency and wavelet transform is described in Additional file 1). A higher level in WT represents a lower frequency band, which shows a low-frequency component in a temporal signal, e.g., a low-frequency signal trend. When the wavelet transform coefficients are set to zero above a certain WT level, the underlying low-frequency trends are likely to be removed. For simplification, we refer wavelet transform coefficients (for both scaling and wavelet functions in a wavelet transform) as wavelet coefficients in the following sections. After that, an inverse wavelet transform is applied to transform the processed signal back to the time domain. To avoid boundary effects, the first and last 2 s were removed in the processed ECG signal [9]. Equation 3 represents the de-trended ECG signal by applying a WT:

3 $${x}^{\prime}\left[t\right]= IWT\left( WT\left(y\left[t\right]\right),\mathrm{wavelet}\ \mathrm{coefficients}\ \mathrm{at}\ \mathrm{certain}\ \mathrm{levels}=0\right),$$

where *x*^′^[*t*] is the de-trended ECG signal, WT(.) and IWT(.) represents a wavelet transform and an inverse wavelet transform, respectively. The de-trended ECG signal was then normalized based on Eq. 1 to be consistent with the scale of original ECG signal.

### Baseline wander (trend) removal evaluation

The evaluation metric in this study was the mean square error (MSE) that measures cumulated errors between the normalized original ECG data, *x*_*N*_[*t*], and the de-trended data, *x*^′^[*t*], to evaluate the performance of each wavelet transform. The same process was done through each sample for each wavelet transform at specific frequency as shown in Eq. 4. We also reported the overall average MSE for each WT across all the simulated trends.

4 $$MSE=\sum_{t=1}^M\frac{{\left({x}_N\left[t\right]-{x}^{\prime}\left[t\right]\right)}^2}{M},$$

We conducted the experiment using the Matlab Maximal Overlap Discrete Wavelet Transform (MODWT) function on a laptop with i7-7500U CPU 2.7 GHz and 8 GB RAM. Each experiment under a specific trend frequency and wavelet type took around 1.17 s. We chose MODWT as it demonstrated to have several advantages over conventional DWT [10, 11].

## Results

In this section, we showed the evaluation results of each wavelet transform in removing baseline wanders.

### Sinusoidal waves

**Fig. 2:**
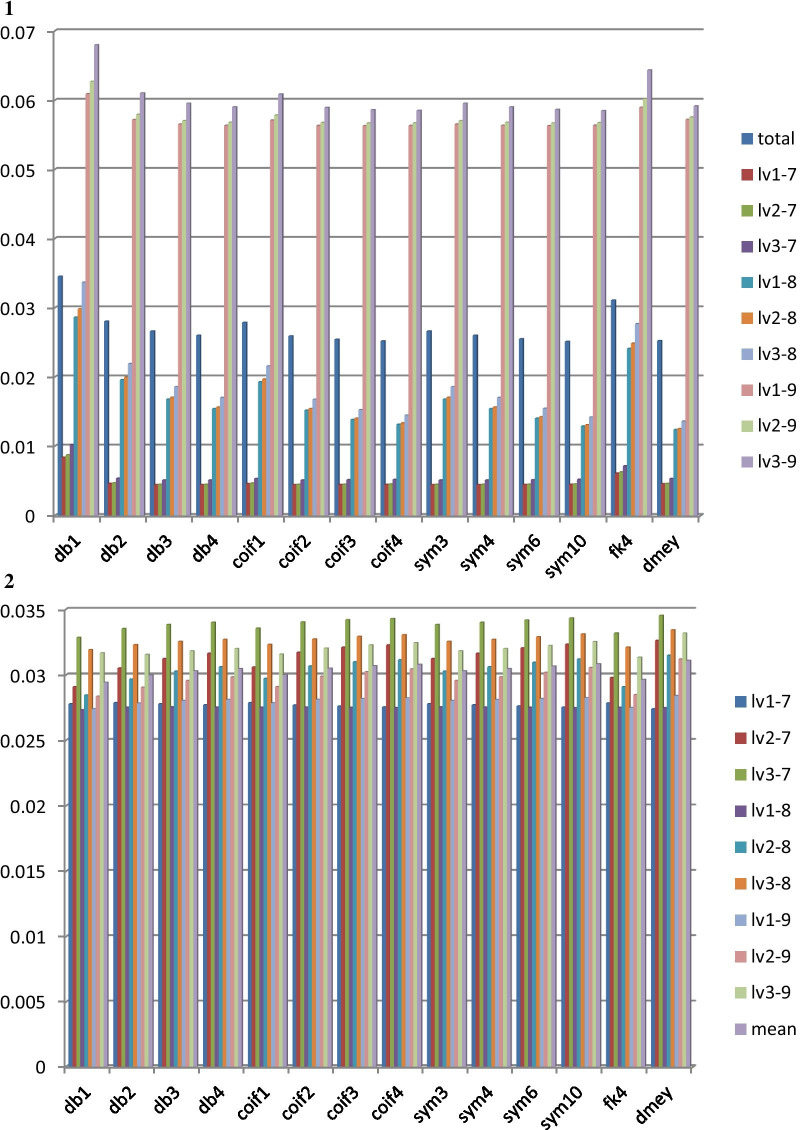

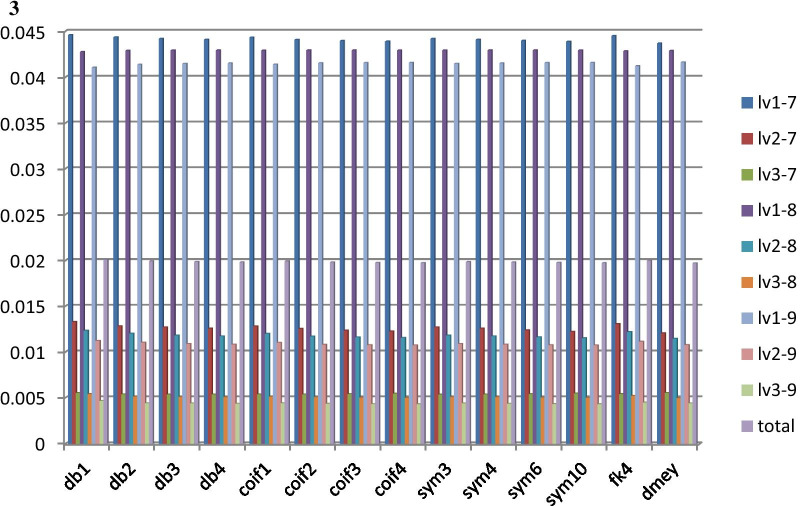
**1**. Sinusoidal Waves Mean Square Error across 14 wavelets: (1) wavelet type:“db”:Daubechies, “coif”:Coiflets, “sym”:Symlets, “fk4”:Fejer-Korovkin, “dmey”:Meyer (2) the result is the average of all trend sinusoidal frequencies (0.05–0.5 Hz). **2**. Step Function Mean Square Error across 14 wavelets: (1) wavelet type:“db”:Daubechies, “coif”:Coiflets, “sym”:Symlets, “fk4”:Fejer-Korovkin, “dmey”:Meyer. **3**. Spike Function Mean Square Error across 14 wavelets: (1) wavelet type:“db”:Daubechies, “coif”:Coiflets, “sym”:Symlets, “fk4”:Fejer-Korovkin, “dmey”:Meyer

**Fig. 3:**
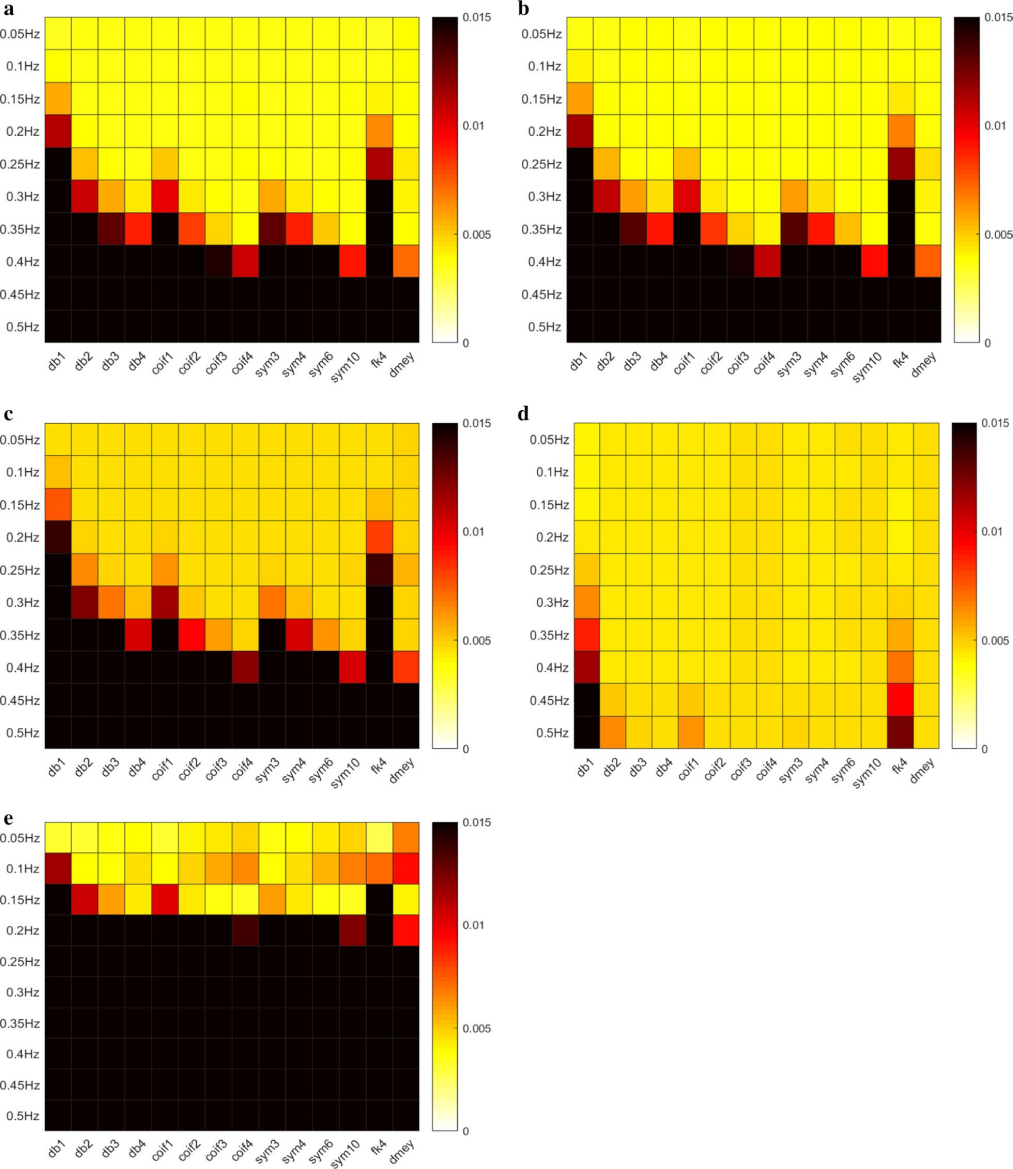
Heatmap of MSEs across different wavelets and frequencies. **a** all the WTs with wavelet coefficients set to zero for levels 9–11 and wavelet coefficients at levels 1 to 8 were preserved (lv1–8); **b** all the WTs with wavelet coefficients set to zero for levels 1 and 9–11 and wavelet coefficients at levels 2 to 8 were preserved (lv2–8); **c** all the WTs with wavelet coefficients set to zero for levels 1–2 and 9–11 and wavelet coefficients at levels 3 to 8 were preserved (lv3–8); **d** all the WTs with wavelet coefficients set to zero for levels 8–11 and wavelet coefficients at levels 1 to 7 were preserved (lv1–7); **e** all the WTs with wavelet coefficients set to zero for levels 10–11 and wavelet coefficients at levels 1 to 9 were preserved (lv1–9)

Figure 2.1 summarized WT performance across different wavelets and different levels of wavelet coefficients being set to zero (for trend removal). Both Daubechies-3 (db3) and Symlets-3 (sym3) had the minimum MSE (0.0044), a mean value across multiple (0.05 Hz–0.5 Hz) simulated trends and over 105 patients in QT database, with wavelet coefficients to be preserved between levels 1 and 7 and wavelet coefficients at the other levels (8–11) to be set to zero (filtered); such process was represented with lv1–7 (Fig. 2). Figure 3 shows heatmaps across different wavelets and wavelet levels to be preserved and filtered. As shown in Fig. 3d, best outcomes across all the wavelets occurred at levels 1–7 with preserved wavelet coefficients and all the other coefficients at levels 8–11 were filtered (setting to 0).

**Fig. 4:**
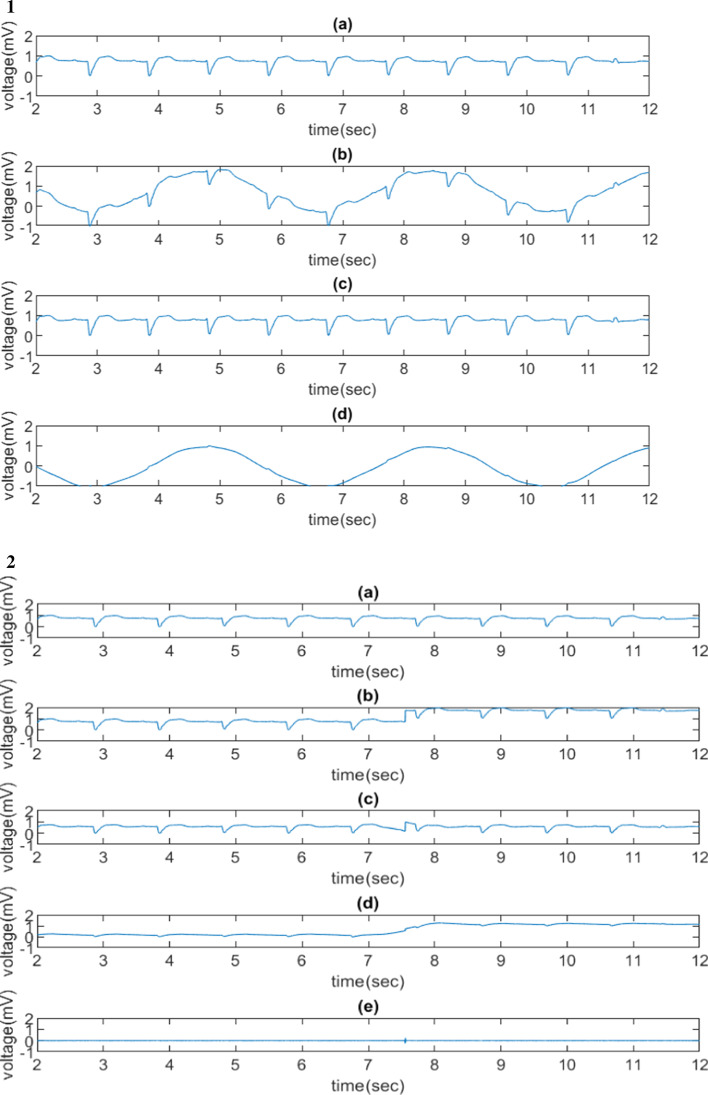

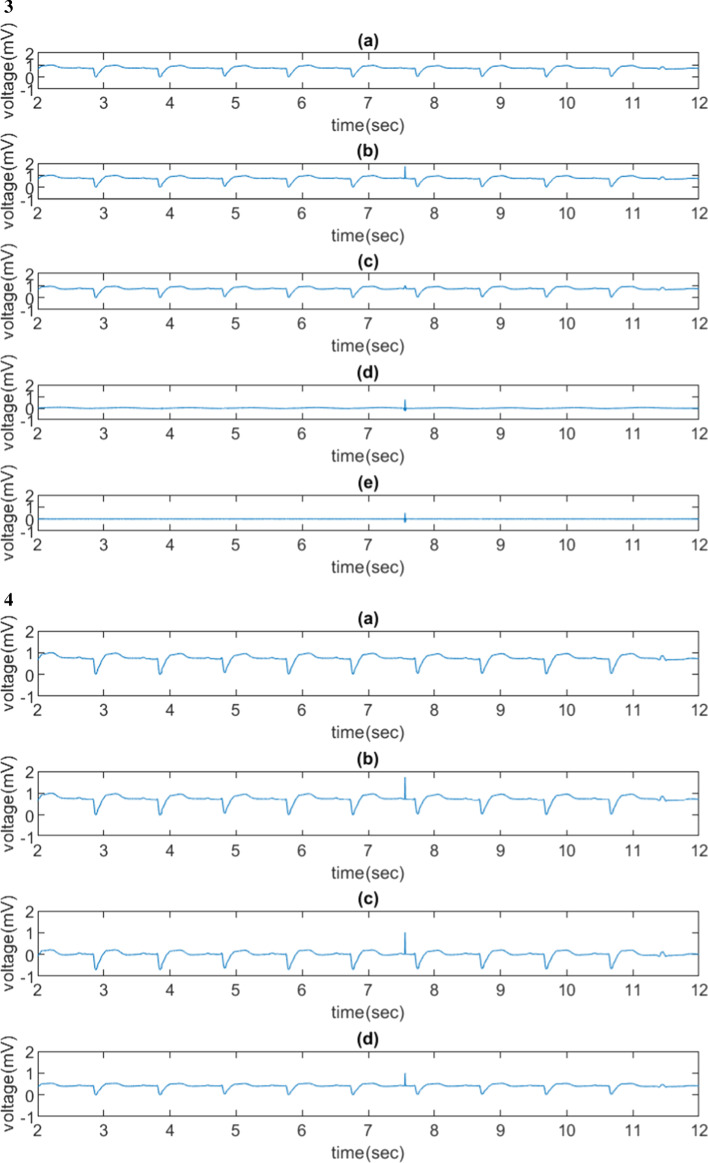
De-trending experiments on one QT database patient (No.sel15814) using sym3 wavelet based on different trends. (1) a trend with a sinusoidal wave at 0.3 Hz added to a normalized raw ECG data (only wavelet coefficients at levels 1–7 were preserved), (**a**) the normalized raw ECG signal, (**b**) semi-synthetic ECG signal formed by superimposing the normalized raw ECG signal and a simulated trend, (**c**) the normalized processed semi-synthetic data with removed trend after applying a WT, (**d**) the extracted trend from (**b**); the MSE between (**a**) and (**c**) was 0.0018 (2) a trend with a step function added to a normalized raw ECG data (only wavelet coefficients at levels 1–7 were preserved), (**a**) to (**d**) following the same process in (1), (e) the reconstructed signal by preserving wavelet coefficients only at level 1 and removing others at other levels; MSE between (**a**) and (**c**) was 0.0337 (3) a trend with a spike added to a normalized raw ECG data (only wavelet coefficients at levels 3–7 were preserved), (**a**) to (**e**) following the same process in (2); MSE between (**a**) and (**c**) is 0.0009, (4) a trend with a spike added to a normalized raw ECG data (only wavelet coefficients at levels 1–7 were preserved), (**a**) the normalized raw ECG signal, (**b**) semi-synthetic ECG signal formed by superimposing the normalized row ECG signal and a simulated trend, (**c**) the non-normalized processed semi-synthetic data with removed trend after applying a WT, (**d**) normalized processed semi-synthetic data with removed trend after applying a WT

Figure 4 shows the de-trending experiments using sym3 wavelet based on different trends. The sym3 with wavelet coefficients at levels 1–7 were preserved and the coefficients at levels 8–11 were set to zero. Figure 4.1 shows the process of forming semi-synthetic ECG data, Fig. 4.1(b), and removing the artificial trend using sym3 wavelet. The artificial trend had a frequency of 0.3 Hz (or 3.3-s cycle). The sym3 wavelet transform accurately removed the simulated baseline wander, Fig. 4.1(c), and the extracted trend was shown in Fig. 4.1(d).

### Spike and step functions

Figure 2.2 and 2.3 summarize WT performance across different wavelets and different levels of wavelet coefficients being set to zero (for trend removal). In the step function experiment, Meyer wavelet had the minimum MSE (0.0274), a mean value over 105 patients in QT database, with wavelet coefficients to be preserved between levels 1 and 7 and set to zero (filtered) between levels 8 and 11. In the spike function experiment, Meyer wavelet had the minimum MSE (0.0044) with wavelet coefficients to be preserved between levels 3 and 9 and set to zero for levels 1–2 and 10–11.

Figure 4.2 to 4.4 show injected artificial trends using a step function and a spike (impulse) function. The injected step-function shown in Fig. 4.2(b) was removed by the WT except during the time between 7 and 8 s. However, the injected spike function in Fig. 4.3(b) was not removed perfectly by the wavelet transform. Figure 4.3(d) shows the location of the impulse edge by preserving wavelet level 1 coefficients and setting all the other coefficients to be zero in the other levels. Figure 4.4(b) was not removed by the wavelet transform.

## Discussion

In this study, we systematically compared 14 wavelets across 10 sinusoidal baseline wanders and two special trends (a step function and a spike function). Most wavelets performed well when wavelet coefficients were preserved at levels 1 to 7 while the rest of the coefficients at levels 8 to 11 were removed (set to 0). Among the 14 wavelets, Daubechies-3 and Symlet-3 wavelet had the best performance; their performance might be attributed by the similarity between their wavelet bases (Daubechies-3 and Symlet-3) and ECG signal [12]. Thus, the two wavelets could potentially better preserve the original ECG signal during signal decomposition. Figure A1 in Additional file 1 shows the wavelet functions for Daubechies-3 and Symlet-3.

The spike-function trend was not removed successfully based on our approach. Since a spike function represents a high frequency signal, the result was expected as our design in this study was to remove low-frequency baseline wanders not the one with a high frequency. However, the high-frequency spike was identified at the time domain by preserving only level 1 wavelet coefficients; an additional removal process could be implemented to remove the spike when the timestamp of the spike is identified by the wavelet transform. In addition, a small MSE (0.0009) was observed, which was attributed by only one sample (spike) point that was not removed among the input ECG signal. In Fig. 2.3, MSE in different WT at level 1 to x preserved, where x ∈{7,8,9}, were unexpectedly large. The reason could be showed in Fig. 4.4. Figure 4.4(b) (semi-synthetic signal) is hardly removed. Since the maximum value of the sample happens to be at the spike point, the normalization step on Fig. 4.4(c) compresses the shape of processed ECG signal. Thus, the MSE is high.

The step-function trend was removed in general but the sudden baseline increase point (within 0.5 s) was not well removed (similar to the spike-trend effect) by the wavelets. Given such short time-period (less than 0.5 s) without completely trend removal, we expected the impact to clinical application would be minimal.

We plan to further compare the two best wavelet transforms (Daubechies-3 and Symlet-3) with other band-pass filters and apply the two wavelets to real-time streaming ECG processing. We expect more accurate ECG analysis such as ST wave deviation can be achieved after applying this wavelet transform-based de-trending process.

## Limitations

In this study, we used simple sinusoidal waves to simulate baseline wanders. In the real world, the baseline wanders could be more complicated. Further research using complicated trends or real-world data are expected.

## Conclusions

We found wavelet transforms in general accurately removed various baseline wanders. Daubechies-3 and Symlets-3 wavelets performed best. The study could facilitate future real-time processing of streaming ECG signals for clinical decision support systems.

## Supplementary Information

**Additional file 1:** Relationship between Sampling Frequency and Wavelet Transform. **Figure A1.** Two wavelet functions: (a) Daubechies-3 and (b) Symlet-3.

## Data Availability

The dataset that supports the findings of this study is available from the public QT database in Physionet [6].

## Abbreviations

ECG: Electrocardiogram
BW: Baseline wander
WT: Wavelet transform
DWT: Discrete wavelet transform
MODWT: Maximal overlap discrete wavelet transform
IWT: Inverse wavelet transform
MSE: Mean square error
db: Daubechies
coif: Coiflets
sym: Symlets
fk: Fejer-Korovkin
dmey: Meyer
lv: Level
