# Supplementary material for: Comparing different wavelet transforms on removing electrocardiogram baseline wanders and special trends
Source: BMC Med Inform Decis Mak. 2020 Dec 30;20(Suppl 11):343. doi: 10.1186/s12911-020-01349-x (PMC7772919; doi:10.1186/s12911-020-01349-x)
Supplement: Supplementary file 1 — Additional file 1: Relationship between Sampling Frequency and Wavelet Transform. Figure A1. Two wavelet functions: (a) Daubechies-3 and (b) Symlet-3. [file 12911_2020_1349_MOESM1_ESM.docx]

**Appendix:**

Relationship between Sampling Frequency and Wavelet Transform

Assume an input ECG signal $f(x)$ has N sample points. The function can be expressed as a linear combination of a scaling function $\alpha\left( x \right)$ and a wavelet function $\beta(x)$:

$$f\left( x \right)=\sum_{k=0}^{N-1} c_{k}2^{{-J_{0}}/2}\alpha\left( 2^{-J_{0}}x-k \right)+\sum_{j=0}^{J_{0}} f_{j}\left( x \right),$$

where $f_{j}\left( x \right)=\sum_{k=0}^{N-1} d_{j,k}2^{{-j}/2}\beta(2^{-j}x-k)$, $J_{0}$ is the number of levels of wavelet decomposition, and $c_{k}$ and $d_{j,k}$ are scale coefficients (for scaling functions) and detail coefficients (for wavelet functions). The first sum is the scale approximation of wavelet transform, and $f_{j}(x)$ are the detail components of wavelet transform. $J_{0}$ depends on the number of sample points N of the signal, $J_{0}=floor({log}_{2}N)$. Thus, in our experiments, given the 14 seconds of the ECG signal sampled at 250 Hz (n=3500), the number of levels ($J_{0}$) of wavelet decomposition is 11, $J_{0}=floor\left( {log}_{2}\left( 3500 \right) \right)$.


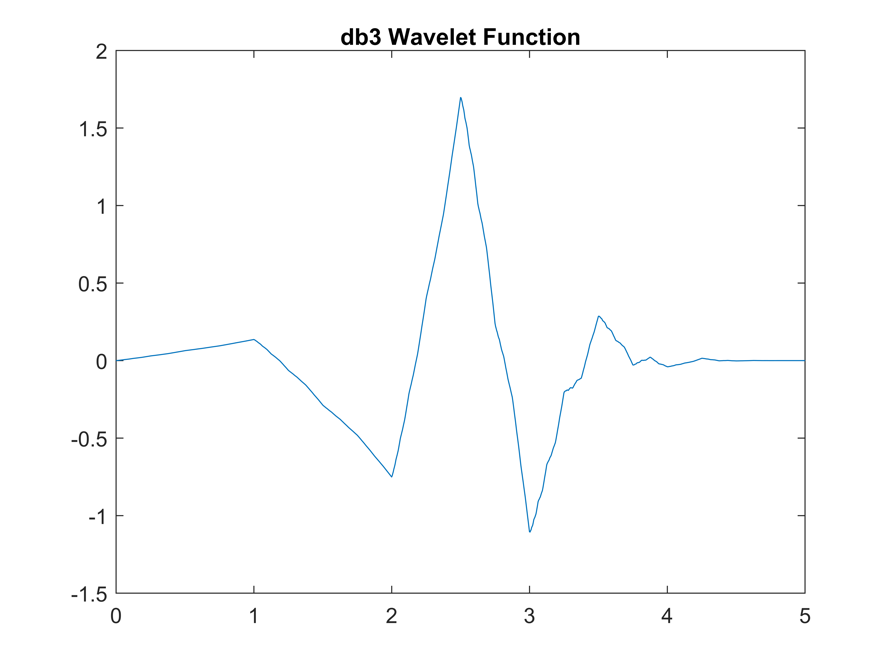

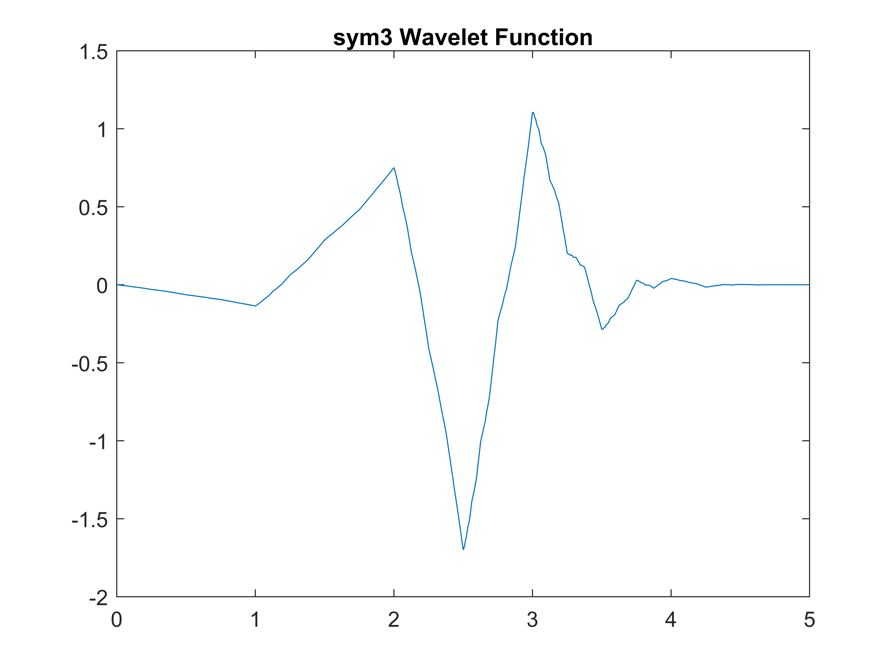


1. (b)

**Figure A1. Two wavelet functions: (a) Daubechies-3 and (b) Symlet-3.**
